# Pupil size and pupil change correlate with distinct cortical neurochemical systems across the adult lifespan

**DOI:** 10.64898/2026.08.24.746786

**Published:** 2026-09-17

**Authors:** Elizabeth Riley, Eve De Rosa, Adam K Anderson

## Abstract

Pupillometry is often used as a proxy for locus coeruleus (LC) activity, but whether this relationship changes across the lifespan is unknown, and important to establish due to the known change in LC biology with age. We collected simultaneous pupillometry and multi-echo fMRI in 89 adults aged 19-78 during a visual oddball task. Oddball-evoked pupillary responses were robust in all age groups but strongest in middle age. Oddball-evoked LC BOLD responses were relatively weak and did not predict individual pupillary responses, so we characterized pupil-BOLD coupling across the whole brain. Pupil size and its first derivative correlated with BOLD on distinct timescales (2.25 s and 5.00 s) and in distinct spatial patterns. Against 19 PET maps, derivative coupling correlated uniquely with norepinephrine transporter (NET) density, and size-unique coupling with μ-opioid (MOR) and cannabinoid CB1 density. Only pupil derivative-specific relationships with BOLD activity (both extent of coupling and association with NET) weakened with age, though both remained significant; other pupil-BOLD relationships did not differ with age. Pupil size and pupil change therefore carry different information about cortical activity throughout the lifespan.

## Full Text Availability

The license terms selected by the author(s) for this preprint version do not permit archiving in PMC. The full text is available from the preprint server.

